# Flow affects the structural and mechanical properties of the fibrin network in plasma clots

**DOI:** 10.1007/s10856-024-06775-1

**Published:** 2024-01-29

**Authors:** Hande Eyisoylu, Emma D. Hazekamp, Janneke Cruts, Gijsje H. Koenderink, Moniek P. M. de Maat

**Affiliations:** 1 Department of Hematology, Erasmus MC, University Medical Center Rotterdam, Rotterdam, The Netherlands; 2 Department of Bionanoscience, Kavli Institute of Nanoscience, Delft University of Technology, Delft, the Netherlands; 3 Department of Biomedical Engineering, Thoraxcenter, Erasmus MC, University Medical Center, Rotterdam, The Netherlands

## Abstract

The fibrin network is one of the main components of thrombi. Altered fibrin network properties are known to influence the development and progression of thrombotic disorders, at least partly through effects on the mechanical stability of fibrin. Most studies investigating the role of fibrin in thrombus properties prepare clots under static conditions, missing the influence of blood flow which is present in vivo. In this study, plasma clots in the presence and absence of flow were prepared inside a Chandler loop. Recitrated plasma from healthy donors were spun at 0 and 30 RPM. The clot structure was characterized using scanning electron microscopy and confocal microscopy and correlated with the stiffness measured by unconfined compression testing. We quantified fibrin fiber density, pore size, and fiber thickness and bulk stiffness at low and high strain values. Clots formed under flow had thinner fibrin fibers, smaller pores, and a denser fibrin network with higher stiffness values compared to clots formed in absence of flow. Our findings indicate that fluid flow is an essential factor to consider when developing physiologically relevant in vitro thrombus models used in researching thrombectomy outcomes or risk of embolization.

**Graphical Abstract:**

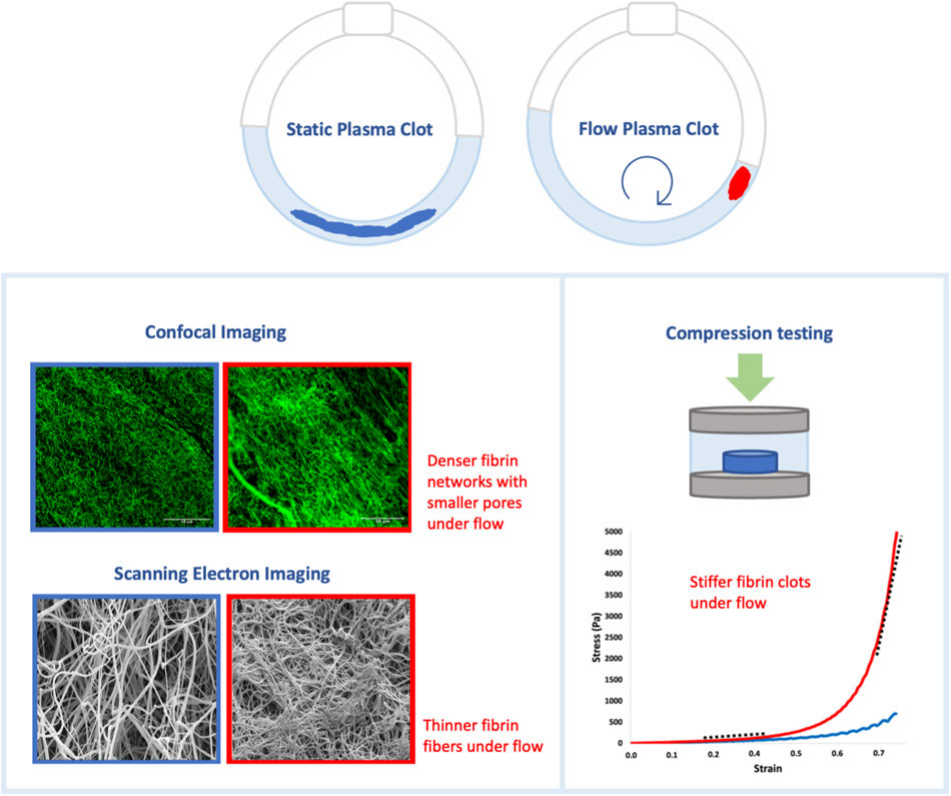

## Introduction

Thrombus formation is the response of the hemostasis system to an injured vessel wall in order to prevent blood loss. When a cerebral blood vessel is occluded by either an embolus or a locally formed thrombus, this can lead to an ischemic stroke, a major cause of mortality and morbidity. Worldwide, there are over 7.6 million new ischemic stroke patients each year, of which about half die [1].

It is still not fully understood how the thrombus contributes to stroke severity and its treatment. The fibrin matrix is an important determinant of the structural integrity and mechanical properties of the thrombus [2–4]. Hence understanding its characteristics may contribute to thrombosis prevention and treatment. Already a number of studies have implicated an abnormal fibrin structure in patients with a thrombotic disease [3, 5–8] with deviations in the structural properties of the fibrin fibers themselves, such as fiber thickness, and of the network they form, such as fiber alignment, density, branch points, and porosity [9]. This likely has clinical consequences because dense matrices with thin fibers and many branch points are more difficult to lyse [10]. Another important aspect of the thrombus are its mechanical properties, since a thrombus needs to be strong enough to withstand the forces due to blood flow and muscle contraction [11]. The mechanical properties of a thrombus determine the risk of rupture and embolization as well as the successful outcome of a thrombectomy, a process that exerts a wide range of forces on the thrombus during retraction or aspiration [11–14]. Therefore, understanding the thrombus architecture and its relation to mechanical properties is essential in understanding the risk of embolization or outcome of treatment [15].

While the fibrin network properties have already been extensively studied [10–12], the majority of these studies were performed under static conditions, whereas in the circulation a thrombus is formed under blood flow [16]. The flow during clot formation influences the clotting process and the fibrin matrix architecture. Thrombi formed in vivo have aligned fibrin fibers, whereas in thrombi formed under static conditions, the fibrin fibers are randomly organized [12, 17]. In addition, it was shown that flow affects fibrin fiber thickness, although studies are not consistent, probably in part because they used different methods [18–20]. Therefore, the effect of flow on fibrin network characteristics is still not fully understood [16, 21]. Similarly, there are a number of studies investigating the mechanical properties of in vitro clot analogs, but the majority of these clot analogs were prepared under static conditions, neglecting the effect of flow [11, 12, 22]. The mechanics of clots formed under flow still remains to be fully investigated [16, 21].

In this study we prepared plasma clots under flow and static conditions to investigate the effect of flow on fibrin network architecture and its mechanical properties. Flow conditions mimicking arterial flow were studied using a Chandler loop setup, where blood or plasma is inserted in tubing that is spun around a rotating cylinder. The rotation of the tubing creates a flow inside the tube in the opposite direction and induces a wall shear stress on the blood or plasma, allowing coagulation in a dynamic environment. We chose the Chandler loop system because it is a convenient, low-tech setup that allows formation of large sized clots under flow which are suitable for thrombectomy studies. Moreover, whole blood clots prepared in a Chandler loop have been shown to resemble arterial clots before [23, 24] and have since been widely used to model thrombi [23, 25–28]. By excluding the cellular components of the clot, we aimed to highlight the influence of the flow condition on the fibrin network alone. Our findings show that flow significantly alters the fibrin network properties and therefore should be incorporated in more physiologically relevant models.

## Methods

### Clot formation in Chandler loop

Citrated blood was collected in 3.2% trisodium citrate tubes (9:1 v/v, Becton Dickinson, Franklin Lakes, NJ, USA) from 3 healthy donors, centrifuged at 2500 g for 10 min at room temperature (rT), then the plasma was mixed and centrifuged again at 2500 g for 10 min to remove platelets and stored at −80 °C until use. This platelet poor plasma (PPP) was supplemented with 0.025 mg/ml AF488-labeled human fibrinogen (F13191, Thermo Fisher Scientific), 6000 pM of Tissue Factor (Siemens Healthineers, Dade Innovin Tissue Factor) and 850 nM CaCl_2_ (final concentrations) and directly transferred to the polyvinylchloride Chandler loop tube (0.3 cm inner diameter and 0.5 cm outer diameter), (Portex) and placed on the rotating cylinder (Stuart Tube Rotator SB3, Stuart) with a diameter of 10.7 cm at a 45° angle. The flow clots were prepared by being rotated at 30 RPM for 90 min and the static clots were prepared by placing the plasma mix on the Chandler loop at 0 RPM for 90 min at room temperature. Six PPP fibrin clots were prepared per flow condition. This rotation rate was chosen since whole blood clots prepared using this setting have previously been shown to resemble arterial thrombi [23, 24].

### Scanning Electron Microscopy (SEM) imaging

A schematic representation of how the clots were sectioned for each measurement is presented in the Supplementary information (Supplementary Fig. 5). Both static and flow clots were sectioned into the head, middle and tail sections and only the middle sections were used for each measurement. Two clots per flow condition were prepared for SEM imaging, with each clot being imaged at high magnification (horizontal field width of 20.7 μm and a pixel size of 7 nm) in at least 5 different, randomly selected regions. To prepare the clots for SEM imaging, they were first washed in cacodylate buffer for 30 min, fixed in 2% glutaraldehyde for 2 h, dehydrated in an increasing concentration of ethanol from 30% to 100% in series, and dried in hexamethyldisilane (HDMS). The dried samples were then secured on aluminum mounting pins (Agar Scientific) by carbon tape (Agar Scientific) and finally sputter-coated with gold (Sputter Coater E5000) to achieve a layer of ~15 nm covering the samples. The SEM images were acquired with a Focused Ion Beam Scanning Electron Microscopy (FIB-SEM) (Helios NanoLab 650). The backscattered ions were detected with an Everhart-Thornley detector at a voltage of 10 kV, with a tilt of 0.0, a dwell time of 3 μs and a working distance of 4.46 mm. The final images were 20.8 × 14.8 μm with 3072 × 2188 pixels.

### Confocal imaging

Four clots per flow condition were imaged using a Leica TCS SP5 Confocal Scanning Laser Microscope (Leica Microsystems, Wetzlar, Germany). The static clots were put on a glass coverslip and the first 12 μm from the bottom of the coverslip was imaged. For each sample, 5 z-stacks were made with a 40x oil immersion lens and the 488 nm laser (4%; gain: 1115). A zoom of 2 was applied, which resulted in images of 193 × 193 μm with 2048 × 2048 pixels. Different settings were used for the flow clots because network features for these denser clots were not distinguishable with the settings for the static clots. Therefore, the first 12 μm was imaged by making five z-stacks with a 63x oil immersion lens and the 488 nm laser (80%; gain: 679). A zoom of 2 was applied, which resulted in images of 123 × 123 μm with 2048 × 2048 pixels. The image speed was 200 Hz for all images.

### Image analysis

The high-resolution images obtained by SEM were only used to quantify fiber diameter, since the sample preparation steps for SEM extensively dehydrate the clots and prohibit mesh size measurements. Meanwhile, the confocal images of hydrated networks were only used to quantify fiber network density and porosity, since the optical diffraction limit prohibited measuring the fiber diameter.

#### Fiber diameter measurement using SEM images

DiameterJ (v1.018) is a validated open-source nanofiber diameter measurement tool suitable for analyzing SEM images available as a plugin for ImageJ [29]. Briefly, raw images were converted to binary images using the segmenting method Statistical Region Merging of order three in DiameterJ. Next, using the thinning algorithm the centerlines of each fiber were found and a Euclidian Distance Map (EDM) was calculated to find the distance between the fiber pixels with the nearest non-fiber pixel. The distance map was then linearly transformed to greyscale. On the greyscale image, the intersection points were found and the radii values within the greyscale values were removed from the center lines. The remaining greyscale values were doubled to convert them to diameter values in μm [29]. A representation of the steps in this workflow applied to an example image can be found in Supplementary Fig. 2 in the Supplementary Material.

#### Pore size and areal fiber density measurement from confocal images

The areal fiber density of the networks was quantified from the confocal images via the percentage of surface area coverage of the fibrin fibers. In ImageJ, image noise was lowered by applying a Gaussian blur as a low pass filter (*σ* = 2). The images were binarized using the Otsu method for automated thresholding. This threshold was chosen after visually testing the provided auto-thresholding methods in ImageJ, which showed that the Otsu threshold filtered the background from the fibers best. The original stacks and the binarized images were added using the operation AND in the image calculator function of ImageJ, to return a stack with all the background noise removed without altering the signal intensity of the fibers. Then, using the ‘analyze particles’ function in ImageJ, the percent surface area coverage of fibers per each slice in a z-stack was measured. A representation of the steps in this workflow applied to an example image can be found in Supplementary Fig. 3.

The porosity of the networks was determined from the same confocal images by adapting the Bubble Code by Molteni et al. [30, 31]. First, the images were binarized in the same manner as for areal fiber density measurements. Then the EDM of the image was calculated, returning the distance to the nearest non-zero pixel (fiber) for each pixel. The local maxima in the EDM represent the coordinates and the radius of a circle that borders fibers. Circles which extended outside of the image were discarded to prevent boundary effects. Additionally, circles with a radius smaller than 3 were removed as observation of the images showed these were beyond the pixel resolution. Molteni et al. [30] proposed criteria for including and discarding circles where the circles are arranged from largest to smallest. The largest circle was chosen as the first to be in the included list. Then, the next circle would be compared to all circles in the included list and added if the center of the smaller circle lies inside one of the larger circles of the included list, meaning that the circles are largely overlapping, and the smaller circle will not be included. This is true when the distance between the centers of the circle is smaller than the maximum between the two circles’ radii. This method allows filtering out largely overlapping circles.

### Compression testing

Four clots per flow condition were prepared for compression testing and two disks of each clot were tested. A schematic representation of how the clots were sectioned for each measurement is presented in the Supplementary information (Supplementary Fig. 5). We measured the macroscopic mechanical properties of the fibrin clots using an in-house unconfined compression tester at Erasmus MC that was previously developed for testing of thrombi by Boodt et al. [13]. It consisted of an aluminum compression plate, attached to a 2.5 N load cell (LSB200 Jr. Miniature S-beam load cell, Futek), which was vertically driven by a linear actuator (EACM4-E15-ZAMK, Oriental motor). During the sample preparation stage, the clots were cut into standard sized disks using a razor and a mold. Here it was much easier to handle the flow clots than the static ones, which were more fragile. Due to this fragility, the static clots were cut into an initial height of 1 mm, while flow clots were 2 mm. The clots were placed on the compression tester stage, which was inside a temperature-controlled basin (37 °C) filled with 4-(2-hydroxyethyl)-1-piperazineethanesulfonic acid (HEPES pH 7.4) buffer. Clots were subjected to 80% compression of their initial height, at a strain rate of 10% per second for 10 loading and unloading cycles. The clot tangent stiffness was calculated from the first 20–40% strain and from the last 75–80% strain of the first loading curve.

### Statistics

Six clots per flow conditions were prepared in total and two of these clots per conditions were prepared for SEM imaging, while the remaining four per condition were prepared for confocal imaging and compression testing. Data is presented as mean and the standard error. A non-parametric Mann-Whitney test was performed using Graphpad Prism 8 to find statistical significance between the static and flow conditions and a *p*-value below 0.05 was considered as statistically significant.

## Results

We investigated the structural and mechanical properties of the fibrin network of plasma clots formed in the presence and absence of flow. We aimed to understand the influence of flow on the structural properties (fibrin fiber diameter, density and porosity) and on the associated clot stiffness. All experiments were conducted on the same pooled plasma in order to eliminate variations between donors.

### Structural characterization of fibrin clots by SEM imaging

**Fig. 1:**
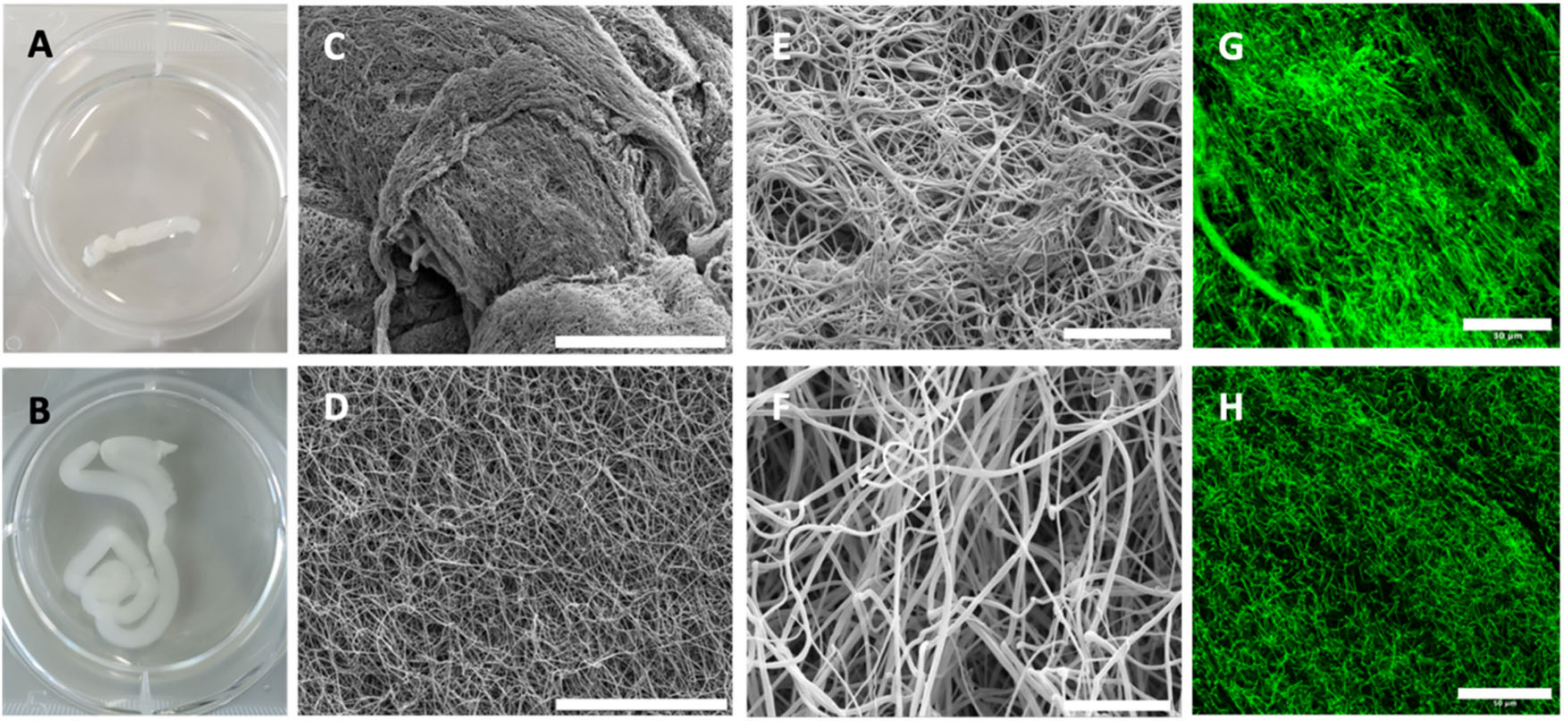
Plasma clots immediately after removal from the Chandler loop tubes: **A** Photograph of a clot formed under 30 RPM flow and of (**B**) a static clot. **C** Low magnification SEM image of a flow clot, showing regions of fibers aligned and twisted in different directions, a range of thicker and thinner fibers and some bundles (scale bar: 40 μm). **D** Low magnification SEM image of a static clot, showing an isotropic and uniform network of fibers (scale bar: 40 μm). **E** High magnification SEM image of a flow clot, showing a dense network composed of thin fibers and bundles (scale bar: 5 μm). **F** High magnification SEM image of a static clot, showing a porous network with fibers that are thicker than for the flow clot (scale bar: 5 μm). Confocal fluorescence images of a flow clot (**G**) and a static clot (**H**), where the green signal comes from fluorescently labeled fibrin. The flow clot shows more fiber alignment compared to the static clot and a brighter fluorescent signal, consistent with a higher density

Interestingly, immediately after clot formation, static and flow clots were clearly distinguishable by eye: the static clots were translucent, longer in size and more delicate to handle (Fig. 1A), while the flow clots were more opaque, shorter in size and sturdier (Fig. 1B). The SEM images showed that the samples were also strikingly different at the network scale. The average length of the plasma clots collected from the Chandler loop tubes were measured and found to be 6.1 ± 0.3 cm for static clots and 1.4 ± 0.2 cm for flow clots, implying a 4.3-fold volume reduction given the constant clot width.

The overview SEM images of the flow clots revealed a more heterogeneous fibrin network when compared to the static clots (Fig. 1C). Some regions had densely packed networks with very thin fibrin fibers while others showed a porous network with thicker fibers (Fig. 1C). We also observed a clear alignment and stretching of fibers in some regions, but it was not possible to observe an overall alignment direction due to the complex flow pattern [32] inside the Chandler loop. The alignment of the fibers in static clots appeared to be isotropic (Fig. 1D). As previously mentioned, whole blood clots prepared under the same conditions have been shown to resemble arterial thrombi with a head and a tail region, showing heterogeneity in fibrin density and cellular component distribution [23, 24]. In order to eliminate such regional variations, we opted to use only the middle sections of the clots obtained from the Chandler loop.

**Fig. 2:**
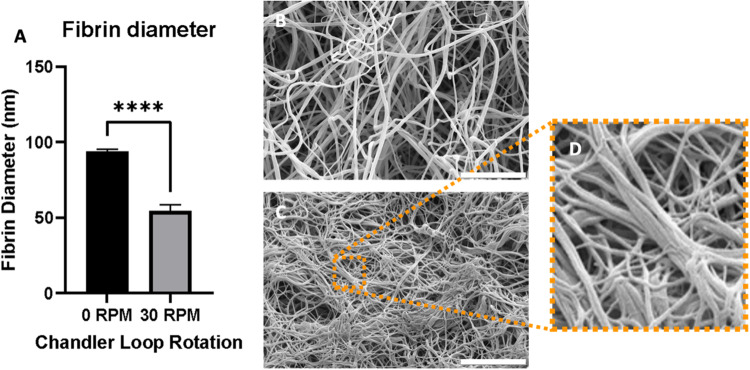
**A** Fibrin fiber diameter measured from high magnification SEM images via DiameterJ reveals that fibers formed under flow (54 ± 4.0 nm) are are significantly thinner than fibers in static clots (94 ± 1.7 nm, *p* < 0.001). High magnification SEM image of a static (**B**) and a flow (**C**) clot used to measure fiber diameter (scale bar: 5 μm). **D** Close-up of boxed region in (**C**) showing that flow clots exhibit coalescing of thinner fibers: multiple fibers are aligned and twisted in the same direction, forming a thicker fiber bundle

Quantification of the fiber diameter using high magnification SEM images showed significantly thinner fibrin fibers in clots formed in the presence of flow (54 ± 4.0 nm, *p* < 0.001) than in clots formed in the absence of flow (94 ± 1.7 nm) (Fig. 2A). In addition to the thinner fibers, we observed more bundled fibers in clots formed under flow (Fig. 2C). As shown in Fig. 2D, thinner fibers bundled and twisted together, appearing collectively as a thicker fiber. The alignment direction in flow clots differed depending on which region of the clot was imaged and thus the direction of the alignment cannot be linked to the direction of flow, even though all the images were taken in the same orientation.

### Confocal imaging

**Fig. 3:**
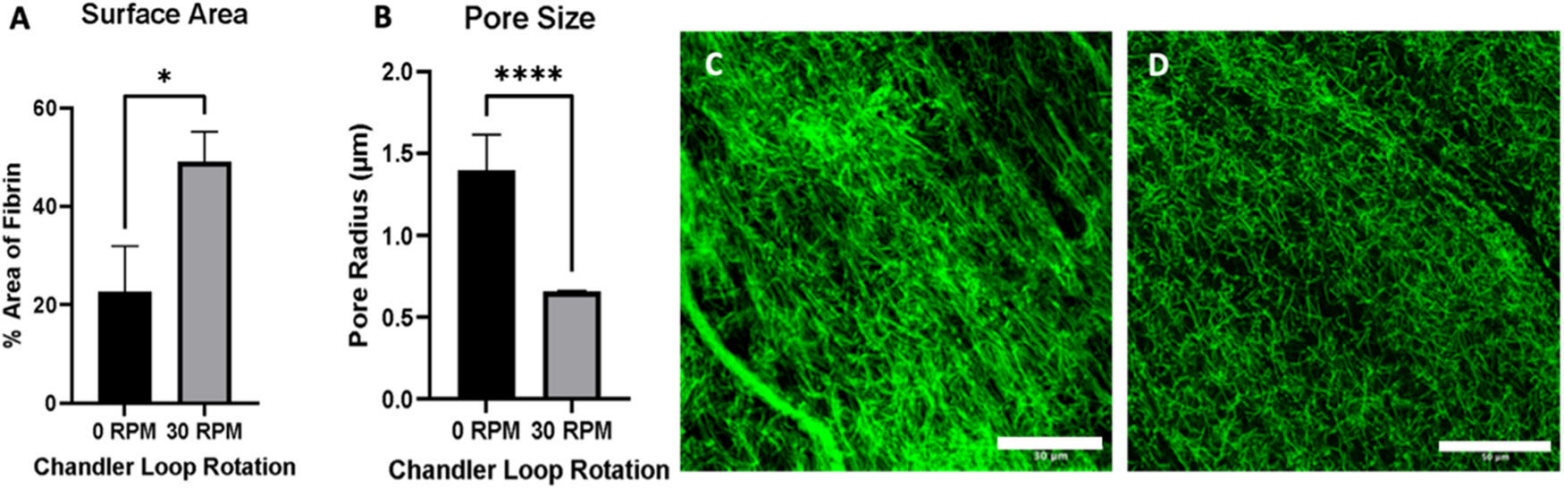
**A** Fractional surface coverage (in %) by fibrin (green) in confocal images was used as a measure of fibrin density. Clots prepared under flow were denser compared to static ones with an average fiber area of 22.6% for static and 49% for flow clots. **B** Confocal images were also used to quantify the network porosity by pore size. In line with the density findings, the average pore radius was significantly smaller in the flow clots compared to the static clots (0.65 ± 0.01 μm versus 1.40 ± 0.22 μm, *p* < 0.001). Confocal images of flow (**C**) and static clots (**D**). Scale bars: 30 μm for (**C**), and 50 μm for (**D**)

We complemented the SEM imaging, which requires sample fixation and drying, with confocal imaging, which has the advantage that we can observe the hydrated network and quantify fibrin density and porosity. The strong fluorescence signal coming from the flow clots prevented a clear observation of the fibers when using the same settings as the static clots, and therefore new settings were applied. These images appeared hazier compared to images of the static clots, where a clearer fibrin network was observed (Fig. 1G, H and Fig. 3C, D). The surface area covered by the green fluorescence was measured as a method to quantify the areal density of the fluorescently tagged fibrin fibers. The clots formed in the presence of flow were significantly denser compared to the static ones, with an average fiber area fraction of 22.6% for static and 49% for flow clots (Fig. 3A). The average pore radius was significantly smaller in the flow clots compared to the static clots (0.65 ± 0.01 μm versus 1.40 ± 0.22 μm, *p* < 0.001) (Fig. 3B). These findings of a denser, less porous flow clot microstructure were in line with the initial gross appearance of shorter, less translucent flow clots.

### Mechanical characterization of fibrin clots

**Fig. 4:**
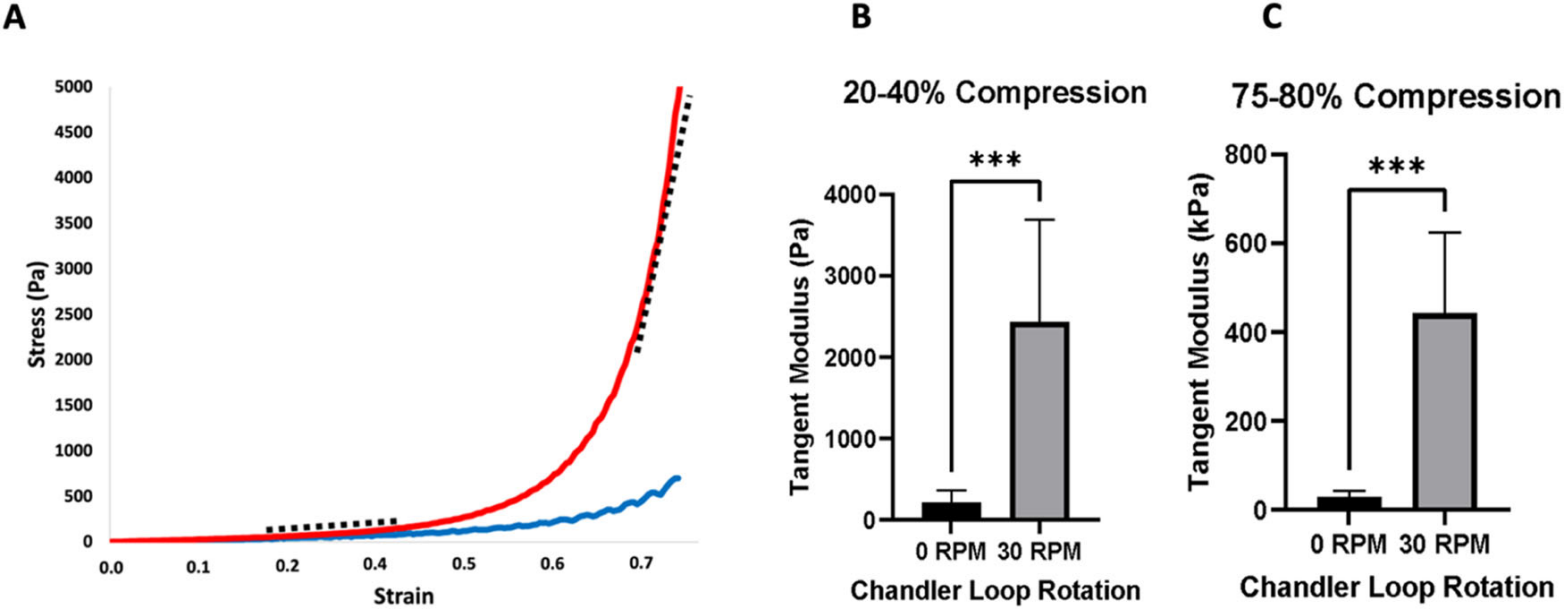
**A** The first loading curve of the compression cycle for flow (red) and static (blue) clots. Both clot types show strain stiffening. The tangent moduli were determined at low strain (20–40%) and at high strain (75–80%), as represented with the black dashed lines on the red curve, for both flow conditions. **B** In the low strain regime (20–40%), the tangent modulus of flow clots is higher (2.4 ± 1.3 kPa) compared to that of static clots (0.21 ± 0.15 kPa, *p* < 0.001). **C** In the high strain regime (75–80%), the tangent modulus of flow clots is again higher (440 ± 180 kPa) compared to that of static clots (29 ± 15 kPa, *p* < 0.001)

The mechanical properties of the fibrin clots were studied using an in-house unconfined compression tester [13] with clots subjected to 80% compression of their initial height. During the compression test, all samples, regardless of flow condition, showed stiffening behavior at higher strains (Fig. 4A). The clot tangent stiffness was calculated from the first 20–40% strain and the last 75–80% strain of the first loading curve. The first 20–40% was chosen to represent the first linear elastic response of the material, while the final 75–80% of the curve represents the stiffening of the material due to high strains. Since thrombi can undergo various levels of strain in vivo and during procedures like thrombectomy, both low and high strain regimes were studied. For both strain regimes, the flow clots were significantly stiffer compared to the static ones with an increase of average tangent modulus from 0.21 ± 0.15 kPa to 2.4 ± 1.3 kPa (20–40% strain, *p* < 0.001) and 29 ± 15 kPa to 440 ± 180 kPa (75–80% strain, *p* < 0.001) (Fig. 4B, C, respectively). During the compression test, ten loading and unloading cycles were recorded for each sample. Regardless of the flow condition, the loading-unloading curves did not superpose, demonstrating a small viscous component to the mechanical response. From the first to the second cycle, the area enclosed between the loading-unloading hysteresis curves decreased, pointing to some network plasticity (Supplementary Fig. 4). After the second cycle, there was no further significant change in the area enclosed by the hysteresis curve between consecutive cycles.

## Discussion

We observed stiffer, denser plasma clots with thinner fibrin fibers when formed under flow in a Chandler loop as compared to static clots. In this study we used pooled PPP for both structural and mechanical characterization. This helped identify the influence of flow on the fibrin network in the absence of cells and eliminated any donor plasma related variations. We showed that flow produced stiffer, denser clots with thinner fibrin fibers as compared to static conditions. In addition, we observed an increase in bundling of fibers when formed under flow. Furthermore, we linked the structural changes in the fibrin network to the stiffness of the clot.

### Influence of flow conditions

We prepared flow clots in a Chandler loop at a rotation rate of 30 revolutions per minute. This setting has previously been used in multiple studies using whole blood and has been reported to yield clots that structurally resemble retrieved arterial clots [23, 24, 33, 34]. Head and tail regions of these whole blood clots showed similar compositional heterogeneity as in vivo thrombi [23]. However, this heterogeneity was not observed in our work comparing the microstructure of fibrin clots from their head, mid, and tail sections, as can be seen in the Supplementary Figure (Supplementary Fig. 1). Although during clot formation we observed that the head region started to form first, with the mid and tail sections growing afterwards, there were no differences in the network density, fiber alignment, or fiber thickness between these sections (Supplementary Fig. 1). Others did describe differences in whole blood thrombi, therefore we selected the mid region for all our measurements. The difference between our plasma clots and reported whole blood clots is most likely explained by the absence of cells in our clots, which are responsible for rearranging and redistributing the clot architecture in vivo [17, 35].

Since the clots formed in the Chandler loop reflect the complex flow patterns inside the tube [32], it was not possible to relate the direction of flow with the alignment of fibers we observed, nor to accurately translate the rotation speed of the Chandler loop to a shear rate value which would correspond to a known arterial or venous flow rate. In vivo, at arterial curvatures and bifurcations or under pathological or stenotic conditions, the blood flow can be turbulent with secondary flow patterns [16, 33, 36, 37]. Thus, the flow pattern inside the Chandler loop with its curvature and secondary flow patterns likely represents these flow patterns where thrombus formation is possible [37, 38]. Additionally, Zeng et al. [28] studied the effect of tube diameter and revolution rate on the clot analog morphology in a Chandler loop system and concluded that it is possible to obtain clots with different structural characteristics by tuning these parameters. The Chandler loop allows preparation of blood clots of various sizes under different flow conditions, which makes it a useful tool for testing out thrombectomy devices and their interactions with different thrombus specimens.

### Fibrin matrix morphology

The fibrin matrix we observed via the SEM images of the flow clots resembled the structure observed previously in patient thrombi [17]. Specifically, we saw regions with varying fiber orientation and density, along with regions with thinner or thicker fibers that showed bundling, similar to the observations reported in patient thrombi. This structural similarity between patient arterial thrombi and the fibrin network in our plasma clots emphasizes the important influence of flow on the fibrin network. We observed fiber alignment in some regions but could not pinpoint a gross alignment direction. Chernysh et al. [17] reported that they were able to observe a gross alignment direction for the overall ex vivo thrombi only in a few cases and this alignment was in the direction of the flow. This structural heterogeneity and fiber alignment was not observed in static clots.

### Fibrin fiber diameter

Fiber diameter is one of the most common parameters used for quantifying the fibrin network characteristics as it is linked to clot stiffness and lytic properties. In our study we showed that thinner fibers formed in the presence of flow. This finding is in line with the findings of Onasoga- Jarvis et al. [35] and Neeves et al. [18], even though they used different methods based on microfluidic devices [18, 39]. Both studies likewise reported thinner fiber diameter with increasing flow rate. Their fiber diameters measured in clots made with a wall shear rate of ~500 s^−1^ were similar to the average fiber diameter values of our flow clots. Similar to our study, they observed more fiber alignment with increased shear [39].

Hethershaw et al. [40] used a Chandler loop to prepare fibrin clots with and without FXIII to observe the effect of this crosslinking enzyme on the fibrin network morphology. They used a similar flow setting to our model and found an average fiber diameter of 74 nm. This value is higher than our average diameter values, but this could be due to the manual [40] versus automated (this work) methods of diameter quantification. We measured fiber diameters using the ImageJ plugin DiameterJ. This method was chosen to eliminate any bias that may arise from manual measurements and has been shown to correlate well with manual measurements for monodisperse networks [41]. However, DiameterJ tends to underestimate the average fiber diameter in polydisperse networks, such as our clots [41]. Campbell et al. [19] reported increasing fiber thickness with increased shear rates by flowing plasma over a coverslip at a 30° angle to induce flow. While this is in apparent contradiction with our results, it should be noted that Campbell et al. [19] mention that, in their flow clots, they observed thin fibers bundling together and they measured these bundles as one thick fiber instead of individual thinner fibers. Instead, we measured the diameters of the individual fibers. Similar to us, Campbell et al. [19] also reported that their flow clots showed more of this bundling behavior than the static ones. Furthermore, fibrin diameters for static clots were similar to our study, with an average diameter of 79 nm [19]. We therefore consider our findings consistent.

### Matrix density and porosity

Independent automated analysis of the fiber density and the average pore size in confocal images showed that, in the presence of flow, fibrin clots were denser and at the same time had smaller pores. This finding is consistent with the initial impression from the gross clot structure (Fig. 1A, B), where the flow clots appeared more opaque and more compact. In the study by Neeves et al. [20], at a given flux, they observe a change in fibrin network morphology from a 3D gel of discernable fibrin fibers to a dense mat of thin fibers and then to a dense fibrin film with undistinguishable fibers with increased shear. A transition from a network with distinguishable fibers to a dense mat structure with thinner fibers could suggest a denser network. The method of sample preparation, thickness of the prepared fibrin clots and the method of induced flow is different for both studies, which could account for this difference in observed network morphology.

### Fibrin clot stiffness

We studied the mechanical properties of fibrin clots under compressive loading when formed under flow versus static conditions. We found that the flow clots, which were denser and had thinner fibers, were also significantly stiffer compared to the more porous static clots that had thicker fibers. All clots showed strain stiffening, minor viscous behavior characterized by hysteresis between the loading and unloading curves, and plasticity characterized by a decrease in the viscous energy dissipation following the initial loading and unloading cycle, regardless of the flow condition (Supplementary Fig. 4). This decrease in energy dissipation following the first cycle was also observed in Liang et al. [42], who made SEM images of plasma clots before and after compression and reported a rearrangement of the fibrin fibers via bundling and criss-crossing due to the compression after the initial cycle, the fibrin networks appeared denser. The remodeling and stiffening of fibrin clots under compression has also been previously shown by Vos et al. [43] and Kim et al. [44], and explained in terms of bond formation between the fibrin fibers when they come in close contact under compression [43]. Kim et al. [45] reported an increase in fibrin network density and fibrin node density, as a measure of branching, with increased compressive strain [45]. Vos et al. used optical tweezers to measure the interactions between individual fibrin fibers and identified new bond formation as a cause of clot stiffening [43]. The bundled, criss-crossed and denser networks were then found to be stiffer, which was also the case in our flow clots when compared to the static ones. Chernysh et al. [46] reported the same behavior in emboli under cyclic testing.

In the high strain regime, the flow clots were about 15 times stiffer compared to the static clots. This stiffness difference is probably mainly due to the difference in clot density. In case of uniform disordered networks, the stiffness of a fibrin network is expected to scale with the square of its concentration [47, 48]. From the gross size difference between static and flow clots collected from the Chandler loop tubes, we estimate that flow clots were ~4 times smaller than the static ones. Assuming the fibrinogen-to-fibrin conversion is the same in both clots, the 15-fold stiffness increase between the two conditions is indeed consistent with this concentration difference. An additional factor contributing to the enhanced rigidity of the flow clots may be the thinner fibers. Fibrin clots prepared using higher thrombin concentrations form highly branched networks of thin fibrin fibers have previously been shown to be stiffer compared to coarse networks with thicker fibers [49]. Additionally, Li et al. reported that as the fibrin diameter of a single fiber increased its stiffness decreased [50].

### Limitations of the study

In vivo, blood clots undergo a wide range of external forces from their surroundings and internal forces due to platelet-driven clot contraction [51]. We investigated the stiffness of fibrin clots under compression, but for a more detailed understanding of the blood clot mechanical properties further mechanical characterization methods varying in types and length scale of loading should be considered for future work. A limitation of this study is that the plasma clots were prepared at room temperature as opposed to body temperature due to the available Chandler loop setup. The use of frozen plasma could additionally influence the fibrin characteristics. Additionally, in vivo, arterial thrombi form under pulsatile flow, which is not captured in the widely used Chandler loop model. For a more physiologically relevant blood clot analog under flow, incorporation of the cellular components is required and of interest for future work. Here, we were able to show the influence of flow on the fibrin matrix alone, but the presence of platelets and red blood cells should cause clot contraction and reorganization, which will in turn influence the clot mechanics [35, 51].

## Conclusion

In this study, we investigated the structural and mechanical properties of the fibrin network when formed in the presence of flow. We showed that the presence of flow significantly alters the network properties by increasing the network density and stiffness while decreasing fiber thickness and network porosity. Therefore, flow should be incorporated for more physiologically relevant blood clot models used in researching thrombectomy outcomes or risk of embolization.

## Supplementary Information

Supplementary Info
