# Supplementary material for: Flow affects the structural and mechanical properties of the fibrin network in plasma clots
Source: J Mater Sci Mater Med. 2024 Jan 29;35(1):8. doi: 10.1007/s10856-024-06775-1 (PMC10824866; doi:10.1007/s10856-024-06775-1)
Supplement: Supplementary file 1 — Supplementary Info [file 10856_2024_6775_MOESM1_ESM.docx]

**Supplementary Information**


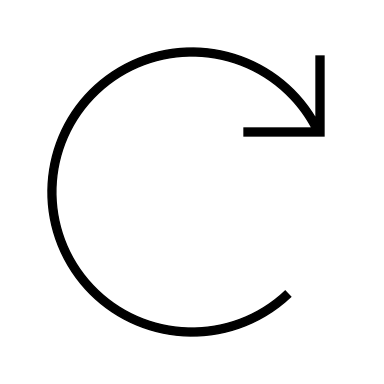


**H**

**T**


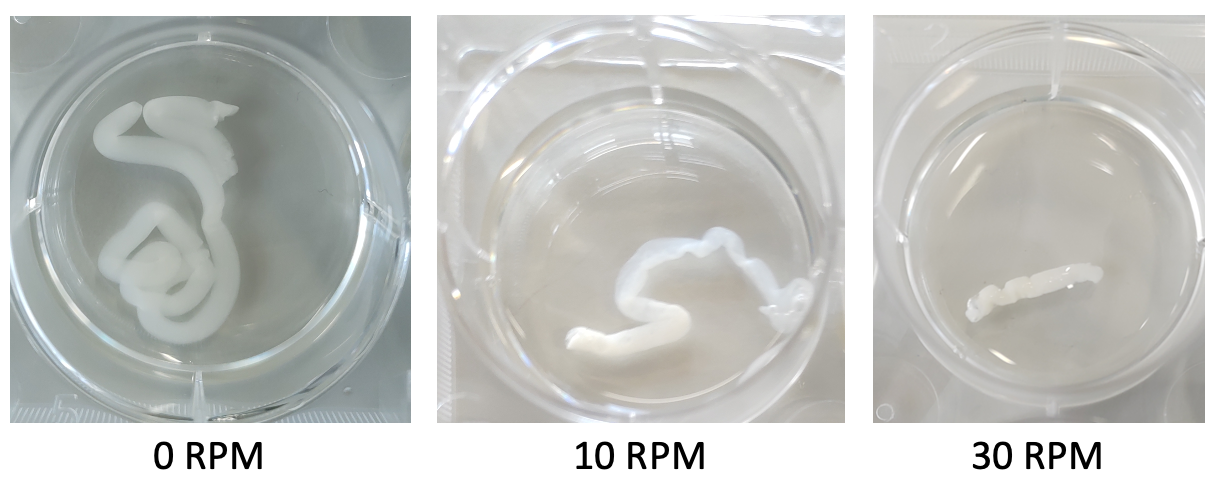


**H**

**T**


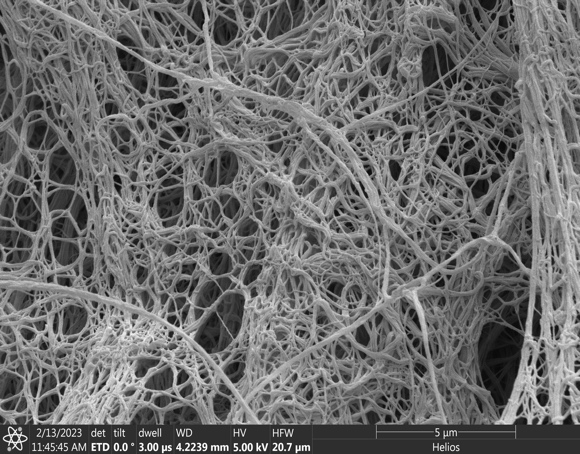

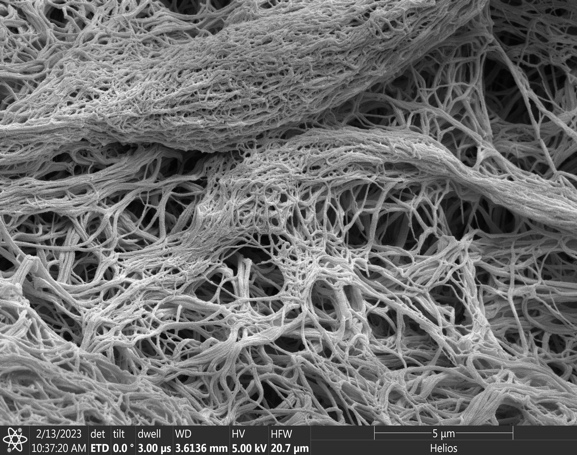


**A**

**B**

**C**

**Supplemental Figure 1:** A) A schematic of the flow clots inside the Chandler Loop: the head region starts to form first at the forward meniscus of the liquid-air interface. On the right: a photo of a flow clot immediately after removal from the Chandler Loop, showing the head (H) and the tail (T) regions. In previous studies using whole blood, a structural heterogeneity was shown between the head and the tail regions of Chandler Loop thrombi, which is representative of in vivo thrombi. B-C) Representative SEM images of the head (B) and the tail (C) regions of a flow clot (scalebar: 5 μm). There were no significant differences between the head and the tail region in terms of the fibrin fiber thickness or density.


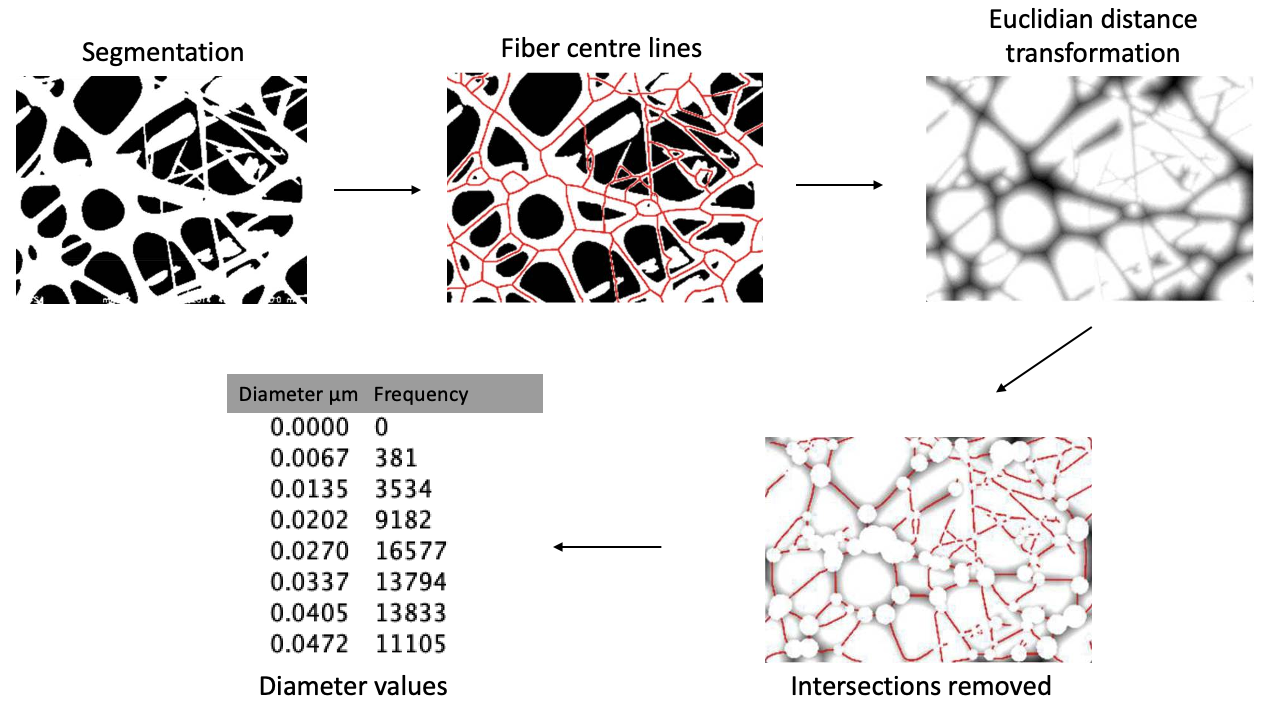


**Supplemental Figure 2:** DiameterJ as an ImageJ plugin was used to calculate fiber diameters from SEM images using. Following the arrows starting on the left: after segmentation of the image, the fiber center lines are obtained. The image is then transformed into a greyscale Euclidian distance map. Then by overlaying the center lines and the greyscale Euclidian distance map, the fiber intersections are removed from the center lines and the remaining lines are used to calculate the diameter values.


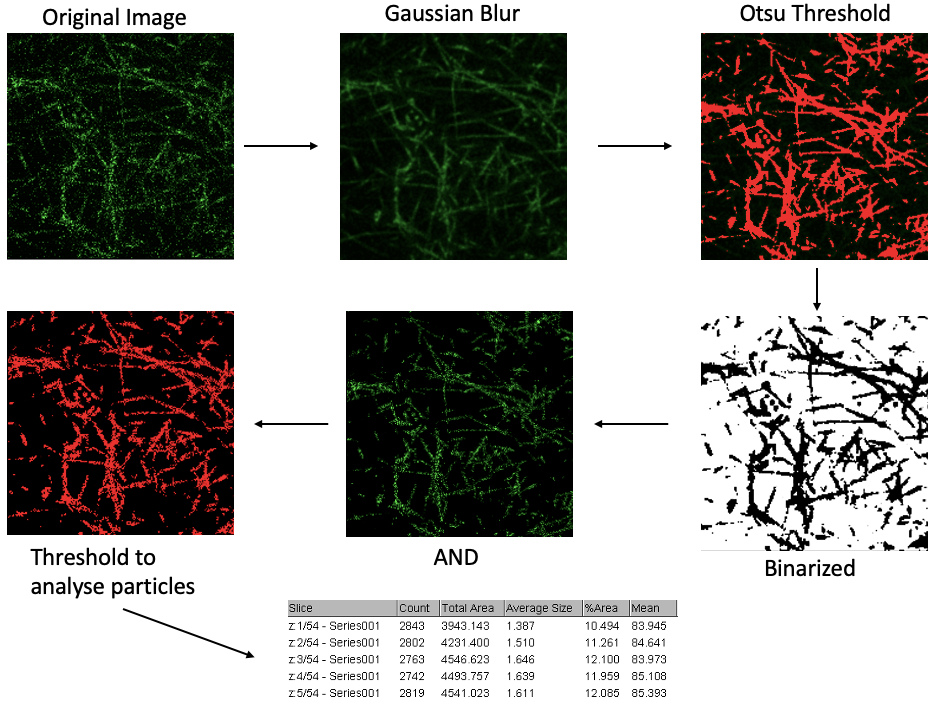


**Supplemental Figure 3:** From the confocal images, using ImageJ, the percentage of fibrin fiber surface area coverage is measured. Following the arrows starting on the left: a duplicate of the original image is treated with a Gaussian blur, then thresholded with Otsu and converted to a binary image. The original image and the binary duplicate are added with the image calculator operator AND, which removes all the background noise in the image but retains the original fiber intensity. Then the image is thresholded and finally the surface coverage of the fibers is extracted.

**B**

**A**


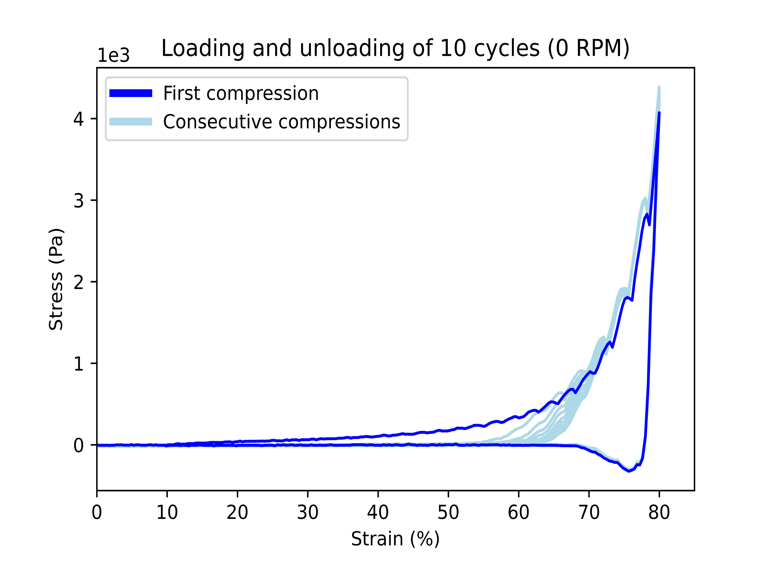

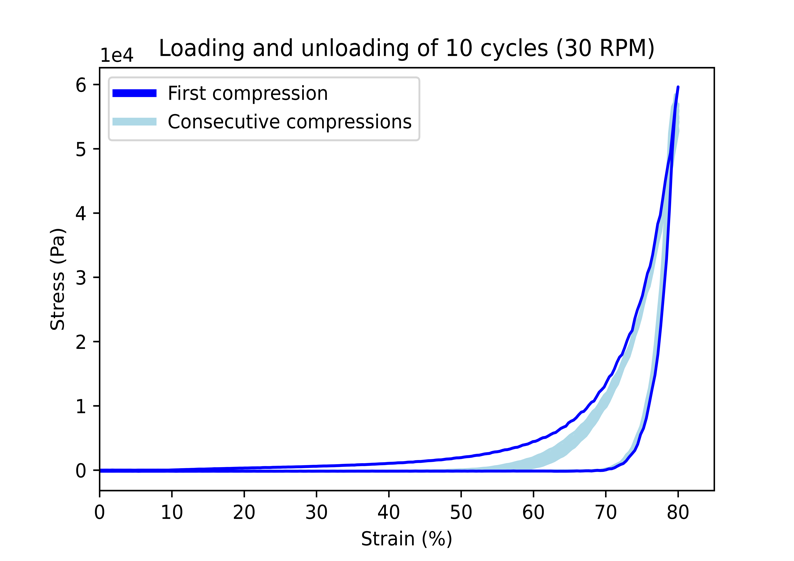


**Supplemental Figure 4:** Representative loading-unloading curves of a flow (A) and a static (B) clot. Both clots were compressed for 10 consecutive cycles. The first cycle is displayed in dark blue and the nine consecutive cycles in light blue. The area under the curve is a measure for viscous energy dissipation during the compression test. Both flow and static clots show a decrease in viscous energy dissipation after the first cycle (dark blue curve). Due to the similarity of the consecutive cycles, individual lines for each cycle cannot be distinguished. The area under the curve for the second (and subsequent) cycles is smaller than for the initial cycle. Some static clot samples adhered to the compression plate which resulted in the below zero values seen in the hysteresis curves (B).

**
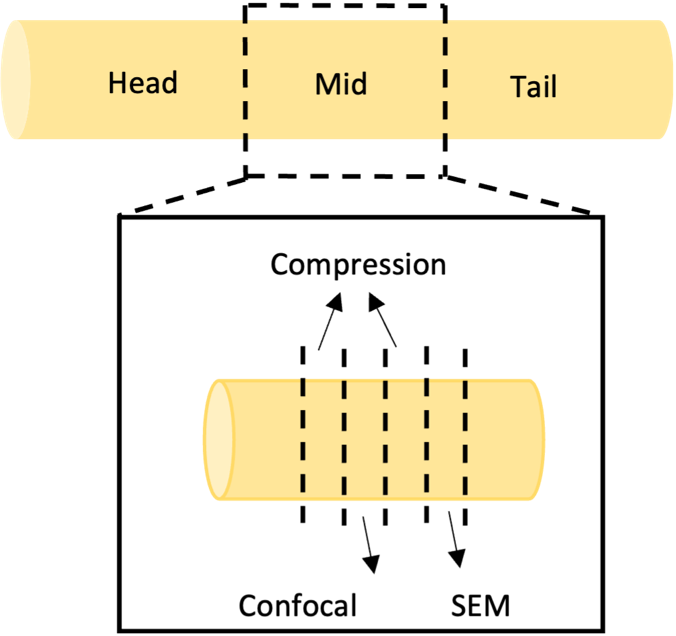
**

***Supplemental Figure 5:*** *A representative figure of a plasma clot labeled by its head, mid, and tail regions. All measurements (confocal, SEM, and compression testing) were conducted on cross-sections taken from the middle section, as shown in the figure.*
